# Nutrition and physical activity programs for obesity treatment (PRONAF study): methodological approach of the project

**DOI:** 10.1186/1471-2458-12-1100

**Published:** 2012-12-21

**Authors:** Augusto G Zapico, Pedro J Benito, Marcela González-Gross, Ana B Peinado, Esther Morencos, Blanca Romero, Miguel A Rojo-Tirado, Rocio Cupeiro, Barbara Szendrei, Javier Butragueño, Maite Bermejo, María Alvarez-Sánchez, Miguel García-Fuentes, Carmen Gómez-Candela, Laura M Bermejo, Ceila Fernandez-Fernandez, Francisco J Calderón

**Affiliations:** 1 Department of Health and Human Performance, Faculty of Physical Activity and Sport Sciences-INEF, Technical University of Madrid, C/Martín Fierro 7, Madrid, 28040, Spain; 2 Department of Physical Education, School of Education, Complutense University of Madrid, C/Rector Royo Villanova sn, Madrid, 28040, Spain; 3 Francisco de Vitoria University, Ctra. Pozuelo-Majadahonda Km 1.800, Madrid, 28223, Spain; 4 Institute of Veterinary Physiology, University of Zurich, Winterthurerstrasse 260, Zurich, 8057, Switzerland; 5 Center for Integrative Human Physiology (ZIHP), Winterthurerstrasse 190, Zurich, 8057, Switzerland; 6 Department of Medical and Surgery Sciences, School of Medicine-IFIMAV, Marqués de Valdecilla Research Institute, Cantabria University, Avda. Cardenal Herrera Oria sn, Santander, 39011, Spain; 7 Nutrition Department, IdiPAZ, University Hospital La Paz Research Institute, Paseo de la Castellana 268, Madrid, 28043, Spain; 8 Laboratorio de Fisiología de Esfuerzo, Facultad de Ciencias de la Actividad Física y del Deporte-INEF, C/Martín Fierro 7, Madrid, 28040, Spain

**Keywords:** Overweight, Obesity, Caloric restriction, Exercise, Weight loss

## Abstract

**Background:**

At present, scientific consensus exists on the multifactorial etiopatogenia of obesity. Both professionals and researchers agree that treatment must also have a multifactorial approach, including diet, physical activity, pharmacology and/or surgical treatment. These two last ones should be reserved for those cases of morbid obesities or in case of failure of the previous ones. The aim of the PRONAF study is to determine what type of exercise combined with caloric restriction is the most appropriate to be included in overweigth and obesity intervention programs, and the aim of this paper is to describe the design and the evaluation methods used to carry out the PRONAF study.

**Methods/design:**

One-hundred nineteen overweight (46 males) and 120 obese (61 males) subjects aged 18–50 years were randomly assigned to a strength training group, an endurance training group, a combined strength + endurance training group or a diet and physical activity recommendations group. The intervention period was 22 weeks (in all cases 3 times/wk of training for 22 weeks and 2 weeks for pre and post evaluation). All subjects followed a hypocaloric diet (25-30% less energy intake than the daily energy expenditure estimated by accelerometry). 29–34% of the total energy intake came from fat, 14–20% from protein, and 50–55% from carbohydrates. The mayor outcome variables assesed were, biochemical and inflamatory markers, body composition, energy balance, physical fitness, nutritional habits, genetic profile and quality of life. 180 (75.3%) subjects finished the study, with a dropout rate of 24.7%. Dropout reasons included: personal reasons 17 (28.8%), low adherence to exercise 3 (5.1%), low adherence to diet 6 (10.2%), job change 6 (10.2%), and lost interest 27 (45.8%).

**Discussion:**

Feasibility of the study has been proven, with a low dropout rate which corresponds to the estimated sample size. Transfer of knowledge is foreseen as a spin-off, in order that overweight and obese subjects can benefit from the results. The aim is to transfer it to sports centres. Effectiveness on individual health-related parameter in order to determine the most effective training programme will be analysed in forthcoming publications.

**Trial registration:**

ClinicalTrials.gov NCT01116856

## Background

The increase in overweight and obesity rates is a major public health concern. In most countries it has affected all population groups regardless of sex, age, race, income or education level, but to varying extents [1]. Based on comparative studies with other European countries, Spain is at the forefront [1], one in four people is overweight or obese, ie 12.5 million people [2]. In fact, two out of three men are overweight and one of every six is obese, with a clear trend to increase in the forthcoming years [3].

Today it is accepted that obesity has a multifactorial origin [4,5] and is a known risk factor for numerous health problems, including high cholesterol, diabetes, hypertension, respiratory problems, musculoskeletal diseases, cardiovascular diseases, and some forms of cancer. Mortality also increases sharply once the overweight threshold is crossed [6,7].

Research has been very active in the last years trying to identify the most effective treatment for adult overweight and obesity [8-40]. A combination of caloric restriction with an increase in energy output by means of exercise has been proven in the hospital setting [41]. Results have been quite conclusive regarding aerobic exercise [9] and very restrictive caloric intake (less than 50% of kcal intake) [35]. But data are still scarce regarding the effect of a combination of strength and aerobic training and a much lesser restrictive hypocaloric diet on weight loss [35]. The PRONAF study wants to get deeper into the latter, also considering the Consensus Paper 2011 from the Spanish Society of Obesity Research (SEEDO), that has stated that the primary goal of treatment should not focus on achieving the ideal body weight, but to have smaller weight losses maintained in the long term [42].

Therefore, the main aim of the PRONAF study was to discover which is the most effective training protocol in combination with caloric restriction for overweight and obese subjects included in a specific intervention program. The aim of this paper is to describe the design and the evaluation methods used for the PRONAF study.

## Methods/design

### Study design

PRONAF is a 22 week-intervention study performed among Spanish overweight and obese adults, who were randomly assigned to one of the following intervention groups: strength training group (S), endurance training group (E), combined strength plus endurance training group (SE), and physical activity recommendations group (C), which are described in detail below. The study was performed twice, one year apart, first in the overweight group (year 2009/2010) and then in the obese group (year 2010/2011). In total, we had 8 interventions groups.

### Population and sample size estimation

The estimation of the sample size was calculated to detect a main effect of the treatment on the percentage of body fat with a 80% statistical power at 5% significance, assuming a 0.80 correlation between repeated measures [43]. The initial calculated sample size per intervention group (n = 22) permits the detection of a large effect size (Cohen’s d = −0.8), as observed in a previous investigation [43].

The initial adjustment of sample size for dropouts carried out used a maximum of 25% of dropouts in each group [37]. Adjusted dropouts sample = n (1/1-R), where n = number of subjects not lost, and R = expected proportion for dropouts. With our data, the initial necessary sample size was: 88 (1 / 1–0.25) = 118 subjects or 29/30 in each intervention group, this being applicable for both the overweight and the obese interventions groups. A total of two hundred thirty nine people initiated the study, completing it 180 (75.3%), which supposed 59 dropouts for different reasons. For personal reasons 17 (28.8%), low exercise adherence 3 (5.1%), low diet adherence 6 (10.2%), job change 6 (10.2%), and lost interest 27 (45.8%).

### Recruitment of the subjects

**Figure 1:**
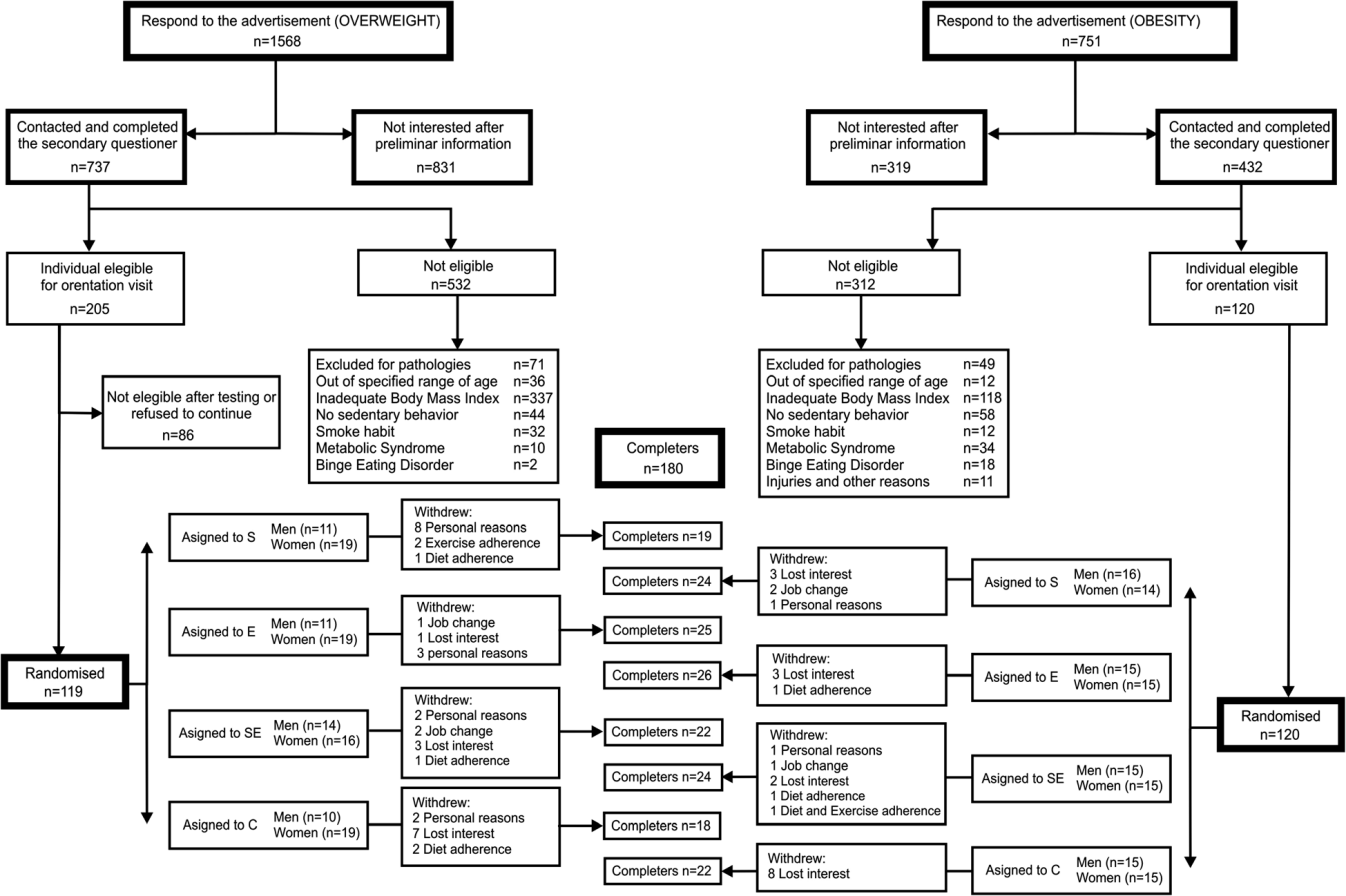
**Participant flow diagram.** S, strength training group; E, endurance training group; SE, combined strength plus endurance training group; C, physical activity recommendations group.

Subjects were recruited by means of diverse advertisements published in the media and the applicants filtered using different criteria. The voluntary subjects who fulfilled the inclusion criteria and passed the baseline physical examination were stratified by age ranges and body mass index (BMI), and randomized into the different intervention groups (Figure 1).

### Inclusion and exclusion criteria

Inclusion criteria included being male or female living in the Region of Madrid, being overweight (BMI 25–29.9 kg/m^2^) or obese (BMI: 30–34.9 kg/m^2^), middle-aged (from 18 to 50 years), having a sedentary lifestyles (< 30 min Physical Activity/day), being normoglycaemic, and non-smoker [37,41]. In the case of females, having regular menstrual cycles was required.

The exclusion criteria were suffering from a physical (orthopaedic limitations, stroke, etc.) and/or psychological (anorexia, bulimia, etc.) disease that may have precluded the performance of the requested strength or endurance training, intake of any medication (beta blockers, alcohol, etc.) known to influence physical performance or the interpretation of the results, having a history of systematic strength or endurance training (moderate to high intensity training more than once a week) in the year before the beginning of the study [37,40].

After the intervention period, participants who failed to comply with 90% assistance to the training sessions and less than 80% adherence to the diet were excluded from further analysis. The characteristics of the final sample (completers) are shown in Table 1.

**Table 1:** Baseline data (n = 180)

	**Overweight n = 84**		**Obesity n = 96**	
	**Women**	**Men**	**Women**	**Men**
	**n = 48**	**n = 36**	**n = 48**	**n = 48**
Age (years)	37.29 ± 8.25	37.42 ± 8.02	39.02 ± 7.74	38.79 ± 7.99
Body Weight (kg)	73.5 ± 5.87	88.21 ± 8.13	88.33 ± 10.1	102.04 ± 8.94
Height (m)	1.62 ± 0.06	1.75 ± 0.07	1.64 ± 0.07	1.77 ± 0.06
BMI (kg/m^2^)	28.01 ± 1.32	28.57 ± 1.12	32.41 ± 1.85	32.4 ± 1.9
Body fat (%)	43.28 ± 3.62	33.8 ± 4.63	47.11 ± 3.49	38.17 ± 4.02
Body fat free mass (kg)	40.25 ± 4.3	55.99 ± 5.47	44.3 ± 4.71	58.63 ± 8.96
Bone mineral density (g/cm^2^)	1.18 ± 0.1	1.26 ± 0.09	1.21 ± 0.11	1.3 ± 0.1

Results are shown as Mean ± SD.

*Note*. BMI = Body Mass Index. Body fat, Body fat free mass and Bone mineral density calculated by Dual-energy x-ray absorptiometry (DXA).

### Ethical issues

The protocols and procedures of the PRONAF study were in agreement with the ethical guidelines on biomedical research on human subjects of the World Medical Association’s Declaration of Helsinki (1964) and further amendments. Before participating in this research, all subjects were carefully informed about the possible risks and benefits of the project, being required to read and sign an institutionally approved informed consent document. PRONAF study was approved by the Human Research Review Committee of the University Hospital La Paz (PI-643).

Access to the database was restricted to the researchers that participated in the PRONAF study. Therefore, the data and information obtained in the project was considered as confidential following current Spanish legislation regulating personal data protection (Organic Law 15/1999 and Royal Decree 1720/2007).

This trial was registered at clinical trials.gov as NCT01116856. http://clinicaltrials.gov/.

### Intervention

#### Time line intervention

The assessments took place for all subjects one week before and after 22 weeks of intervention. Additionally, body weight was measured every 15 days, training intensity and physical activity were controlled monthly, and dietary intake was evaluated every three months.

#### Exercise protocols

Four different intervention groups were involved in the study as stated above. The S, E and SE groups followed the corresponding training program plus the dietary intervention, and the C group followed the dietary intervention and was instructed about the general recommendations regarding physical activity for weight loss from the American College of Sports Medicine (ACSM) [44].

**Figure 2:**
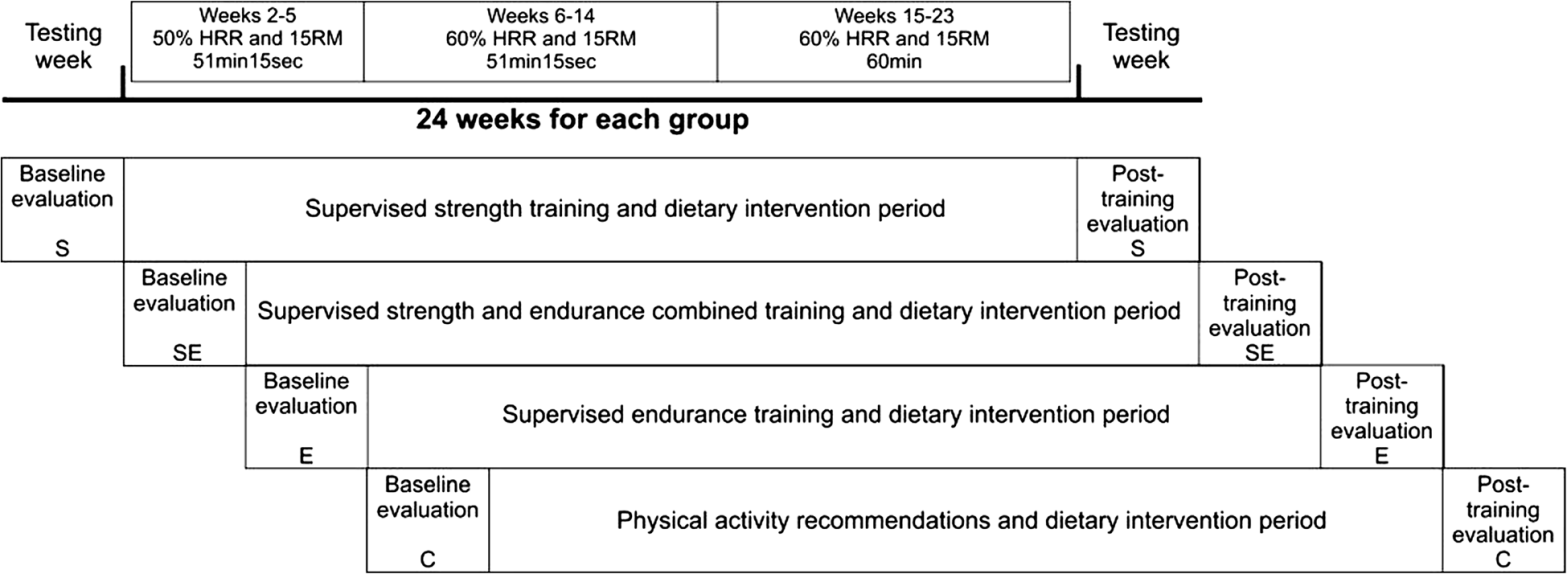
**Time line of the Study.** S, strength training group; E, endurance training group; SE, combined strength plus endurance training group; C, physical activity recommendations group.

Subjects in the S, E and SE groups performed training 3 times/wk for 22 weeks at a Sports Centre in Madrid (Spain). All training sessions were carefully supervised by certified personal trainers. The exercise programs were designed taking into account each subject’s muscle strength (MS) and the heart rate reserve (HRR). MS was measured using the 15-repetition maximum (15 RM) testing method [45] in the S and SE groups (both of which involved strength training). Both volume and intensity of exercise was increased over the study period (Figure 2) according to standard procedures [35,41].

Prior to each training session each subject started with a 5-minute aerobic warm-up routine, followed by the session routine, and concluded with a 5-minute cool down and stretching exercises routine. Moreover, an MP3 audio track was played for each participant to set the execution rhythm of the exercises during all the sessions. In this case, the heart rate (HR) was monitored by a pulse-meter (Polar RS800CX, Polar Electro, Kempele, Finland). The cadence for the resistance exercises was fixed at 1:2 (concentric-eccentric phase). A session routine consisted in the execution on eight scheduled exercises in the case of group S (i.e., shoulder press, squat, barbell row, lateral split, bench press, front split, biceps curl and French press for triceps). Regarding types of exercises, running, cycling or elliptical (self-selected) exercises were the main components of the session routine for group E, while the routine for the SE group consisted in a combination of cycle ergometry, treadmill or elliptical intercalated with squat, row machine, bench press and front split.

The volume and intensity of the three training programs increased progressively. During the adaptation period (cf. weeks 1–4) subjects were taught on the exercise technique. Between the fifth and eight week, exercises were carried out at an intensity of 50% of 15RM and HRR, performing 2 laps to the circuit (cf. 51 minutes and 15 seconds), then between the 9^th^ and 14^th^ week the intensity was increased up to 60% of 15RM and HRR, and finally during the period between the 15^th^ and 24^th^ week the volume increased from 2 to 3 laps to the circuit (64 minutes). Then, a five-minute recovery period was set between the circuits. The S and SE participants performed 15 repetitions (45”) for each exercise with a rest period of 15 seconds between them.

#### Diet intervention

Hypocaloric diets (between 5028 and 12570 KJ) were prescribed individually for all participants by expert dieticians at the Department of Nutrition, La Paz University Hospital, Madrid. The diet was designed to provide 25-30% less energy than the daily energy expenditure (DEE), as measured using SenseWear Pro3 Armband™ (Body Media, Pittsburgh, PA). Approximately, 29–34% of the energy came from fat, 14–18% from protein, and 50–55% from carbohydrates [46]. In the obesity group, energy provided from protein was increased up to 14–20%.

### Before-after measurements

#### Physical fitness and health

##### Maximal aerobic power test

The test was conducted on a computerized treadmill (H/P/COSMOS 3P^®^ 4.0, H/P/Cosmos Sports & Medical, Nussdorf-Traunstein, Germany). Peak oxygen uptake (VO_2_ peak) was measured with the modified Bruce protocol used elsewhere with overweight and obese population [35,47]. Volume and composition of expired gas was analysed with a gas analyzer Jaeger Oxycon Pro (Erich Jaeger, Viasys Healthcare, Germany) which validity and reliability has been demonstrated previously [48,49], and continuous 12-lead electrocardiographic monitoring. The exercise test was maintained until exhaustion. The mean of the three highest measurements was used as VO_2_ peak. Identical procedure was carried out to calculate the maximal HR.

#### Resting metabolic rate

Subjects were asked to refrain from any exercise for at least 24 h before the test. To assess the resting metabolic rate (RMR), participants were cited between 7 am to 10 am, after a 9 h overnight fasted period. RMR was then measured by indirect calorimetry standing up (during 11 min) and in a lying position (additional 20 min). Oxygen uptake (VO_2_) and carbon dioxide production (VCO_2_) were recorded by a gas analyzer Jaeger Oxycon Pro (Erich Jaeger, Viasys Healthcare, Germany), and resting heart rate (RHR) was measured using a heart rate monitor (Polar Electro Oy, Kempele, Finland). All these parameters were averaged during the last 5 min of the standing up position and the last 10 min of the lying position. Oxigen uptake and VCO_2_ data were used to calculate RMR according to the formula of Weir [50].

#### Maximal dynamic strength

Muscle strength was measured with the 15RM testing method described elsewhere [51] for both strength training groups S and SE. The 15 RM for each exercise was tested twice on different days during the evaluation periods. The intraclass correlation coefficient of reliability for all the exercises was ICCr = 0,995 and ICCr = 0,994 for men and women, respectively. All the assessments, the data collection sessions and the previous trainings were carried out with the same machines and free weights (Johnson Health Tech. Iberica, Matrix, Spain).

#### Static strength (dinamometry)

The general strength index (SI) was calculated using previous criteria [52]. This method measures leg and arm strength with respect to body weight via two exercises: the bench press and squat. The dynamometric strength index (DSI) was calculated by measuring muscular strength using a Tecsymp Tkk5002 hand dynamometer and a Tecsymp Tkk5401 leg and back dynamometer (Tecsymp, Barcelona, Spain). The DSI value was calculated as the sum of the values obtained with both apparatuses divided by subject’s body weight.

#### Physical activity and daily energy expenditure

Habitual physical activity (PA) was assessed with a SenseWear Pro3 Armband™ (Body Media, Pittsburgh, PA). Subjects were instructed to wear the monitor continuously for 5 days including weekend days and weekdays following general recommendations [53]. Data was recorded by 15 min intervals. DEE was calculated using the Body Media propriety algorithm (Innerview Research Software Version 6.0).

#### Energy balance, adherence to diet and exercise

All subjects were instructed to continue their habitual daily activities as before the intervention period. They were required to report the kind, duration, and intensity of any physical activity and the amount of any food undertaken during the intervention period.

At the beginning of the intervention, negative energy balance was calculated taking into account the DEE, and a 3-day food record, in order to decrease the energy intake of the diet by a 25-30% during the intervention.

In one hand, adherence to diet was calculated as the estimated Kcal of the diet divided by the real Kcal intake in percentage ([estimated kcal of diet/real Kcal intake] · 100), representing the highest adherence the value 100%. Higher values mean a higher restriction and viceversa. On the other hand, adherence to exercise was calculated by the number of sessions completed in regard to the theoretical sessions ([sessions performed /total sessions] · 100).

#### Psychological and eating behavior assessment

##### Quality of life

The impact of overweight and non-morbid obesity on Health-Related Quality of Life (HRQL) was assessed using two different questionnaires:

#### EUROQOL 5D (EQ-5D)

The EQ-5D, is a self-administered questionnaire capable of generating a score reflecting a value associated with a given health status. The EQ-5D™ descriptive system consists of five dimensions: 1. Mobility; 2. Self-care; 3. Usual activities; 4. Pain/discomfort; 5.Anxiety/depression. Each dimension has three levels: “level 1: no health problems”, “level 2: moderate health problems” and “level 3: extreme health problems”. Each unique health state described by the instrument has an associated 5-digit descriptor ranging from 11111 for perfect health to 33333 for the worst possible state. The resulting descriptive system defines 243 health states. The instrument also includes a 20-centimeter visual analogue scale for the self-assessment of current general health [54].

Short Form-36 Health Survey (SF-36) version 1.4, valid for the Spanish population was used [55]. This questionnaire evaluates the individual health status in two dimensions: functional and emotional. Functional status is divided in 6 subscales: Physical Functioning (PF), Role-Physical (RP), Bodily Pain (BP), Vitality (VT), Social Functioning (SF) and Role-Emotional (RE). Emotional status includes Mental Health (MH) and General Health (GH) subscales. Scores on all subscales range from 0 to 100 (higher scores indicate better health status) [56].

##### Eating behaviour

At baseline and at the end of the intervention all food and beverages consumed by the participants were recorded using a food frequency questionnaire and a “3-day food and drink record”, validated for the Spanish population [57]. This recorded all food and drinks consumed at home and away for three days, including 2 weekdays and 1 weekend day. Subjects were instructed to record the weights of food consumed if possible and to use household measurements (spoonfuls, cups, etc.) if not. The energy and nutritional content of the food consumed was then calculated using the DIAL software (Alce Ingeniería, 2004). Then, the values obtained were compared to the recommended dietary intakes for the Spanish population to determine dietary adequacy [58].

#### Body composition

##### Anthropometric measurements

Body height was measured using a SECA stadiometer (range 80-200 cm, Valencia, Spain). Body weight (BW) was measured using a TANITA BC-420MA balance (Bio Lógica Tecnología Médica S.L, Barcelona, Spain). BMI was calculated as [body weight (kg)/(height (m))2]. Waist circumference (WC) was measured using a SECA 201 steel tape (Quirumed, Valencia, Spain). Body fat percentage was measured using bioelectrical impedance analysis (BIA) with an OMRON BF 306^®^ analyzer (OMRON HEALTHCARE Co., Ltd, Ukyo-ku, Kyoto, Japan).

##### Dual-energy x-ray absorptiometry

Dual-energy x-ray absorptiometry (DXA) was used to measure percentage of total fat mass (TFM%), percentage of android fat (AF%), the android gynoid ratio (A/GF) and lean mass (kg), using a GE Lunar Prodigy apparatus (GE Healthcare, Madison, Wisconsin, USA). All DXA scan analyses were performed at the Faculty of Physical Activity and Sport Sciences using GE Encore 2002 software v 6.10.029.

##### Nuclear magnetic resonance

Nuclear Magnetic Resonance (MRI) was used to study the proportion and location of the adipose tissue. The MRI study measured changes at different abdominal adipose tissue compartments: SAT (superficial and deep depots) and VAT (intraperitoneal and retroperitoneal compartments). Images were obtained with a GE Medical Systems 1.5 Tesla whole body scanner using a T1-weighted fast-spin echo pulse sequence at the University Hospital *La Paz* Research Institute. The subjects were examined in supine position with arms positioned parallel along the lateral sides of the body. The subjects were imaged in two half-body volumes. A breath-hold sequence during expiration (approximately 20 seconds per acquisition) was used to minimize the effects of respiratory motion on the images. The total research time was about 5-10 min. All images were acquired on a 256x192 matrix, 16 bits/pixel, with a field of view = 480 mm. Slice thickness was 6 mm and contiguous slices were obtained every 1.5 mm from de first sacral vertebra upwards, until to achieve 30 images per person. For each subject six reference levels were studied, corresponding to intervertebral disc levels L5-S1 to L2-L3, at umbilical and at caudal left kidney positions. The acquired axial MR images were retrieved from the scanner using the DICOM^®^ protocol and after were transferred to an external personal computer running Windows XP. We used specially designed image analysis software Stereonauta for quantitative analysis of the images.

#### Biochemical, metabolic, inflammatory and genetic profile

##### Biomarkers

Blood samples were taken early in the morning at the La Paz University Hospital Extraction Unit after a 12 h overnight fast by venipuncture from the antecubital vein. Samples were kept at 4- 6°C until analysis, which was always performed within 48 h following standard hospital procedures. The following parameters were measured:

•Hematology was performed in a Pentra 120 DX cell counter (ABX-Horiba, Montpellier, France).

•Serum calcium, phosphorus, total proteins, albumin, creatinine, uric acid, bilirrubin, lipase, alkaline phosphatase, creatinkinase, LDH, GOT and GPT were analyzed by enzymatic-spectrophotometric assay (Olympus AU 5400, Izasa, Porto, Portugal).

•Blood lipid profile (total cholesterol, HDL and LDL cholesterol, triglycerides) and glucose were analyzed by means of a enzymatic-spectrophotometric assay (Olympus AU 5400, Izasa, Porto, Portugal). Apo A and Apo B were determined using a BNII nephelometer (Siemens Healthcare Diagnostics GmbH, Eschborn, Germany). Atherogenic risk ratios were calculated: total cholesterol/HDL, LDL/HDL and Apo B/Apo A1.

•Plasma fasting inmunoreactive insulin was measured by enzymatic method in a fluoro-immunochemistry Analyzer (AIA-360, Tosoh Bioscience, Tessenderlo, Belgium). Insulin sensitivity was estimated using the HOMA-IR index [HOMA-IR = fasting glucose (mmol/l)/fasting immunoreactive insulin (mU/ml)/22.5].

•Inflammatory biomarkers: Plasma C-reactive protein concentrations (CRP) were determined using a BNII nephelometer (Siemens Healthcare Diagnostics GmbH, Eschborn, Germany). Plasma fibrinogen was measured by coagulometric methods (ACL TOP 700, Izaasa, Porto, Portugal). Plasma leptin, IL-6 and TNF-α plasma concentrations were determined using a Luminex^®^-LX200 Analyzer (Millipore Corp, Billerica, Massachusetts, USA) and a MILLIPLEX MAP human circulating cancer biomarker magnetic bead panel (HCCBP1MAG-58 K) (Millipore, St. Charles, Missouri, USA). All samples were analyzed in duplicate. Data were analyzed using 3.1 xPONENT Software (Millipore, St. Charles, Missouri, USA). The intra and inter-assay coefficients of variation for the cytokine assays all fell in the 5-10% range.

•25-hydroxyvitamin D was measured by chemiluminescent immunoassay using the LIAISON” Analyzer family (Palex Medical, Barcelona, Spain).

•Serum TSH was measured by an electrochemiluminescence immunoassay (ECLIA) by using an E170 analyser (Roche Diagnostics, Mannheim, Germany).

##### Genetic profile

We investigated the potential influence of different genetic single nucleotide polymorphisms (SNPs), highly prevalent in the general population, on the response to different training protocols in overweight and obesity treatment. These SNPs are located in genes involved in metabolic pathways related to adiposity (Beta-Adrenergic and Leptin receptors, and Peroxisome Proliferator-Activated Receptor), and in genes involved in energy metabolism and the production of energy during physical activity (metabolism of lactate and fatty acids). In addition, we have studied the Angiotensin-Converting Enzyme (ACE) Insertion/Deletion (I/D) polymorphism, which has been associated with athletic performance and training adaptation. The potential role for most of the selected polymorphisms is well documented, both as overweight/obesity risk factors and as modulators for the response to different interventions.

With regard to the method used, we firstly reviewed published literature about the techniques for analyzing the polymorphisms [59-68]. Then, we adapted these protocols to the conditions in our laboratory. Whole blood (5 ml) from each patient was collected in EDTA and sent to the Metabolism, Genetics and Nutrition Research Group of the School of Medicine-IFIMAV, in Santander. DNA was extracted from each sample using the “QIAamp^®^ DNA Blood Mini Kit” from QIAGEN (Hilden, Germany) and the genotyping was performed for each SNP afterwards. The DNA samples were preserved at −40°C. All subjects signed a specific informed consent which allows storage and genetic characterization in relation to the objectives of the PRONAF study by the Metabolism, Genetics and Nutrition Research Group of the IFIMAV following the protocols of confidentiality and clinical safety, ensuring the anonymity of the samples and their use for research only.

After selecting the most interesting SNPs for the project objectives, the analyses were performed using genotyping assays based on techniques of double PCR, PCR and RFLP or sequencing for Phase II, and real-time PCR (TaqMan^®^ SNP Genotyping Assays, Applied Biosystem) for Phase III, which allows the semi-automation of these determinations. All these techniques proved to be reproducible and stable under the conditions of the study. The reliability of the techniques used for genotyping in the different phases of the study has been proved using internal quality controls through different methods: repeated measurements of randomized samples, double-blinded reading of random samples, introduction of positive and negative controls, and random doubled testing of samples by both techniques. We obtained consistent results for any of the techniques used.

### Assessment during the follow-up

#### Training control

Feedbacks for training loads were done once a month with the Rate of Perceived Exertion (RPE) to subjectively evaluate each session and determine where the participant considered the intensity to be at, following a similar methodology previously used [69].

#### Physical activity control

PA was assessed as described above once a month. All subjects were instructed to continue their habitual daily activities as before the intervention period and were provided with a PA diary to log the type, duration, and intensity of any PA or exercise undertaken during the intervention.

#### Body weight control

BW was measured as described earlier every fifteen days at the Sports Center. Subjects included in group C used their own scales to monitor BW at home during the intervention.

#### Dietary intake control

A dietician interviewed each participant at baseline, and at 3 and 6 months after the start of the intervention. All subjects were instructed about how to record their dietary intake using a daily log, and given recommended portion sizes and information on possible food swaps. In addition, voluntary group nutrition education sessions were given by the dieticians. The goal was to equip the participants with the necessary knowledge and skills to achieve gradual, permanent behavioural changes.

### Statistical analysis

The main independent variables of the study were: intervention group, age, gender, measurement (baseline – post) and BMI classification (overweight or obese). The delta percentage was calculated thought the standard formula: change (%) = [(post-test score − pre-test score)/pre-test score]*100. The power calculated previously to find significant differences between groups was 0.8 to fat mass (%).

Standard statistical methods will be used for the calculation of the means and standard deviation for all dependent variables. The statistical analysis will be as follows: 1) Univariate descriptive analysis, study of data distribution, basic statistics such as central and dispersion values. Pair comparison tests with previous analysis of the homogeneity of variance will be used. Pearson correlation coefficients, chi-squared tests and exact probability calculations will be also performed to study the relationship between quantitative and qualitative variables, respectively; 2) General lineal models, such a multivariate analysis (MANOVA) or two way analysis of variance (ANOVA) for repeated measures, will be used to determine differences among the four interventions groups and between baseline and post-training values. Compound symmetry, or sphericity, will be verified by the Mauchly test. When the assumption of sphericity would not be met, the significance of F ratios will be adjusted according to the Greenhouse-Geisser procedure. Multiple comparisons of ANOVAs will be made with the Bonferroni post -hoc test.

The analysis of the data will be done using statistical package SPSS (SPSS Inc., Chicago, Illinois, USA). The significant level will be set at α = 0.05.

### Assessment for quality control

Previous to the start of the intervention we developed a protocol book with all the tests carried out in the pre and post intervention, plus the intervention procedures. An external observer followed the pilot study and his observations were included in the protocols. Weekly meetings during the pilot study were made with all the people involved in the project to train them and perform the protocol book.

At the end of the overweight intervention, the subjects filled in a satisfaction questionnaire. The results were included in the design of the obese intervention.

Finally, in order to clean the final data base of possible errors, a double data entry procedure was performed. Also an assessment of missing data and the identification of potential outliers were carried out.

## Discussion

The PRONAF study is the first randomized controlled intervention study performed in Spanish overweight and obese adults without any other associated disease with the aim of losing weight and improving several health-related parameters by means of combining caloric restriction and controlled training programs. One of the strengths of the PRONAF study is the multidisciplinary approach. A group of professionals and researchers with expertise in a specific field: physical activity and fitness, nutrition, body composition, genetics and biochemical markers have designed together the study protocols after an in-depth review of the state of the art in the literature [8-40]. Exercise protocols were tested in healthy subjects and study protocols harmonized in a pilot study [70,71].

The PRONAF study has both strengths and limitations. The main limitation is that the study did not include psychological control of the subjects. During the study and in the final questionnaire evaluation the subjects pointed out the need of some psychological support especially when the body weight changes were not so evident.

On the other hand, the PRONAF study has also several strengths: 1) the final sample size of our study was maintained according to the sample size estimation, keeping a low dropout rate similar to other studies [37,40,41]. It is important to note that there were no differences between groups in the number of dropouts. 2) During the training sessions the participants in the study were continuously monitored by certified trainers to ensure the correct execution of the exercises and the completion of each training session. 3) Inclusion of additional variables as visceral fat, blood lipid profile, and genotyping broadens the dimension of the PRONAF study.

In summary, feasibility of the PRONAF protocol has been proven. Transfer of knowledge is foreseen as a spin-off, in order that overweight and obese subjects can benefit from the results. The aim is to transfer it to sports centers. Effectiveness on individual health-related parameter will be analyzed in forthcoming publications.

## Competing interests

The authors declare that they have no competing interests.

## Authors’ contributions

All authors participated in the writing of the paper and provided comments on the drafts and approved the final version.

## Authors’ information

PRONAF Study Group

Coordinator: Benito P.J.

Local clinical treatment teams and researchers (Principal Investigators are bolded, alphabetical order);

*Madrid UPM*: Alvarez-Sánchez M, **Benito PJ**, Bermejo M, Butragueño J, Calderón FJ, Cupeiro R, Díaz V, González-Gross M, Morencos E, Peinado AB, Rojo-Tirado MA, Romero B, Rossignoli I, Torres RM, Valtueña J, Zapico AG.

*Madrid IDIPAZ*: Bermejo LM, Fernández-Fernández C, **Gómez-Candela C**, Zurita, L.

*Santander HUMV*: Amigo T, **García-Fuentes M**, González- Lamuño D, Redondo C.

## Pre-publication history

The pre-publication history for this paper can be accessed here:

http://www.biomedcentral.com/1471-2458/12/1100/prepub
